# Prevalence of lymphedema symptoms across cancer diagnoses and association with depression, pain interference and health-related quality of life

**DOI:** 10.2340/1651-226X.2025.42203

**Published:** 2025-01-21

**Authors:** Gitte Sone Larsen, Christoffer Johansen, Annika von Heymann, Bolette Skjødt Rafn

**Affiliations:** Danish Cancer Society National Cancer Survivorship and Late Effects Research Center (CASTLE), Department of Oncology, Copenhagen University Hospital Rigshospitalet, Copenhagen, Denmark

**Keywords:** Cancer survivorship, cancer-related late effects, cancer-related lymphedema, swelling, heaviness, tightness, patient-reported outcomes

## Abstract

**Background and purpose:**

Lymphedema is a debilitating late effect of cancer treatments, yet its prevalence beyond breast cancer remains understudied. This study examined the prevalence of lymphedema symptoms across cancer diagnoses and their association with depression, pain interference, and health-related quality of life (HRQoL).

**Patients/Material and methods:**

This cross-sectional study was conducted at the Department of Oncology, Copenhagen University Hospital, from February to April 2021, as part of a broader investigation into cancer-related late effects. Here, we present data from patients in follow-up who received online lymphedema symptom assesments (swelling, heaviness, or tightness). Utilized questionnaires were the European Organization for Research and Treatment of Cancer Quality of Life Questionnaire, the Major Depression Inventory, and the Brief Pain Inventory. Associations between lymphedema symptoms and depression, pain interference, and HRQoL were examined via multiple linear regression.

**Results:**

Of 1,901 patients in follow-up who received the lymphedema symptom items, 1,296 responded. Most participants had breast cancer (48%), followed by testicular (17%), gynecological (16%), and head/neck cancer (11%). One-third (*n* = 397) reported lymphedema symptoms, with 38% (*n* = 152) reporting moderate/severe symptoms. The highest symptom prevalence was seen in gynecological cancer (59%), followed by head/neck (41%), breast (21%), and testicular cancer (19%). Participants with moderate/severe lymphedema symptoms were significantly more likely to report higher depression and pain interference scores and lower HRQoL scores compared to those with no/mild symptoms.

**Interpretation:**

Lymphedema symptoms are highly prevalent among patients who have completed treatment for diverse cancers and associated with higher scores for depression and pain interference, and lower HRQoL.

## Introduction

As the global population of cancer survivors continues to rise due to increasing incidence and improved survival rates, recognizing and addressing cancer-related late effects becomes ever more critical [1]. Lymphedema stands out as a debilitating late effect that is frequently overlooked, especially outside the well-studied context of breast cancer [2]. The lack of awareness and understanding of the prevalence of lymphedema across cancer diagnoses hinders early detection and timely treatment for many survivors.

Cancer-related lymphedema arises from a compromised lymphatic system, commonly resulting from anticancer treatments such as surgery, lymph node dissection, and radiation therapy [3]. The condition manifests as swelling due to the accumulation of extracellular fluid in areas adjacent to the treatment site and may present with additional symptoms like heaviness, tightness, and numbness. Early edema can be transient and reversible but may progress to lymphedema, which, once established, can worsen, become chronic and profoundly impact survivors’ lives [4].

Lymphedema is a visible and painful reminder of survivors’ cancer journey, with some describing the swollen limb as a ‘balloon arm’ or an ‘elephant’s leg’ [5, 6]. This not only affects survivors’ self-image and physical function but also causes psychological distress [7, 8]. For survivors of head and neck cancer, lymphedema may cause difficulties swallowing, compromising their health-related quality of life (HRQoL) [9]. Additionally, lymphedema can impose a considerable economic burden, including direct costs for treatment and indirect costs related to reduced work performance and employment opportunities [10, 11].

While research on breast cancer-related lymphedema has advanced prospective surveillance and treatment strategies [12], a startling lack of clinical awareness and research regarding lymphedema remains among survivors of other types of cancer and particularly among male cancer survivors. Several systematic reviews have highlighted this knowledge gap [3, 13, 14], and studies employing consistent methodologies for assessing the prevalence of lymphedema symptoms across various cancer diagnoses are therefore warranted.

This study aimed to examine the prevalence of lymphedema symptoms across cancer diagnoses among survivors who were in follow-up after treatment for cancer and explore the association with depression, pain interference, and HRQoL.

## Methods

### Design and participants

This cross-sectional study was conducted at the Department of Oncology, Copenhagen University Hospital Rigshospitalet, Denmark, from February 2021 to April 2021 as part of a broader investigation into cancer-related late effects. The parent study collected patient-reported outcomes (PROs), socio-demographic and clinical variables from individuals diagnosed with cancer via an online questionnaire, which were linked with clinical data from the medical records. All patients aged ≥18 years with access to the national digital mailbox (‘e-Boks’) who were in active treatment or follow-up after cancer at the department in February 2021 were invited to the parent study. For the current analysis, only survivors who were in follow-up for: (1) bladder, colon, breast, gynecological, head/neck, kidney, prostate, or testicular cancer; and (2) who had completed the lymphedema symptoms items were included. Participants with incomplete lymphedema symptom items, multiple cancer diagnoses, or those in active cancer treatment, except for hormone treatment, were excluded.

### Outcome measures

#### Socio-demographic and clinical characteristics

Participants reported age, sex, body mass index (BMI), cancer diagnosis, time since diagnosis, and treatment history, including surgery, chemotherapy, radiation therapy, hormone therapy, and/or immunotherapy. Participants’ medical records were accessed to extract comorbidity data, which were used to calculate the Charlson Comorbidity Index (CCI).

#### Lymphedema

The primary outcome was lymphedema symptoms defined as heaviness, swelling, or tightness in the neck, arm, hand, breast, legs, groin, or genital region, as relevant for each cancer type. These symptoms were assessed using lymphedema symptom items from the European Organization for Research and Treatment of Cancer Quality of Life Questionnaire (EORTC-QLQ): VU34 (item 39–47), BR23 (item 47–53), and H&N43 (item 64). For instance, one item asked: ‘During the past week, have you had swelling in your neck?’. The response format was a 4-point Likert Scale (1 = ‘not at all’, 2 = ‘a little’, 3 = ‘quite a bit’, or 4 = ‘very much’). Symptoms of lymphedema were defined as moderate to severe if at least one item was scored ≥ 3 [15].

#### Depression

The Major Depression Inventory (MDI) is a validated self-rating scale covering the frequency of 10 depressive symptoms within the last 2 weeks, assessed on a 6-point Likert scale (0 = ‘at no time’, and 5 = ‘all the time’). The total score ranges from 0 to 50, with ≤ 20 indicating no depression, 21–25 mild depression, 26–30 moderate depression, and ≥ 30 severe depression. In this study, depression was defined as a cut-off score ≥ 21 [16].

#### Pain interference

Pain interference in daily activities (general activity, walking, work, mood, enjoyment of life, relations with others, and sleep) was evaluated using the Brief Pain Inventory (BPI) short version. Each item was rated from 0 to 10 (0 = ‘Does not interfere’ to 10 = ‘Completely interferes’), with the final score calculated as the average of the seven items. A score of < 2 indicates no/mild pain interference, ≥ 2–5 moderate pain interference, and ≥ 5 severe pain interference [17]. This study used a cut-off score of ≥ 2 for pain interference.

#### Health-related quality of life

To evaluate HRQoL, this study utilized the five functioning scales (physical, role, cognitive, emotional, and social) and global health status/QOL from the EORTC-QLQ-C30 [18]. The functioning scales use a 4-point Likert scale (1 = ‘not at all’ to 4 = ‘a lot’), and the two items in the global health status/QoL use a 7-point Likert Scale (1 = ‘very poor’ to 7 = ‘excellent’). Scores are linearly transformed to a 0–100 scale [15]. Higher scores on the functioning and global health status/QOL scales indicate better levels of functioning [19].

To identify significant functional impairments and symptom burden, the thresholds of clinical importance, as defined by Giesinger et al. [19], were applied. Scores exceeding these thresholds indicate a potential need for clinical intervention.

#### Charlson Comorbidity index

Participants’ comorbidities were extracted from electronic medical records and used to calculate the 17-condition CCI. Each condition was scored between one and six points, summed and interpreted as 0 = no comorbidity, 1–2 = moderate comorbidity, and ≥ 3 = high comorbidity [20]. In the analysis, comorbidity was dichotomized and reported as absent or present (≥ 1).

### Statistical analysis

Descriptive statistics summarized sociodemographic and clinical variables using frequencies for categorical variables and median and interquartile range (IQR) for continuous variables. The prevalence and severity of lymphedema symptoms were presented descriptively for cancer diagnoses reported by ≥ 5 participants.

Variables associated with moderate to severe lymphedema symptoms across cancer diagnoses were identified using univariate logistic regression. The analysis included only cancer diagnoses with ≥ 100 survey responses. Independent variables included age, BMI, CCI, and treatment modalities. Results were summarized using odds ratios (ORs) with 95% confidence intervals (CIs).

To compare differences in depression, pain interference, and HRQoL between participants with no/mild and moderate/severe lymphedema symptoms, Welch’s t-test and multiple linear regression were used. Results were reported as differences in means (MD) with 95% CI in models that were: (1) unadjusted; (2) adjusted for the sociodemographic variables (sex, age, BMI, and CCI) (Adjusted Model I); and (3) adjusted for sociodemographic and the clinical variables (time since treatment, and treatment modalities: surgery, chemotherapy, radiation therapy, hormone therapy, immunotherapy) (Adjusted Model II). The risk-difference for exceeding the threshold for clinical importance across the five HRQoL functioning scales was compared between participants with no/mild and moderate/severe lymphedema symptoms. Risk differences were reported with corresponding 95% CIs.

Among participants who received the lymphedema symptom items, responders and non-responders were compared using chi-square or Fischer’s exact tests for categorical variables and the Mann-Whitney U test for continuous variables.

### Ethics

The study was approved by the Research Legal Department at the Capital Region of Denmark (reference number: 23039798). All participants provided written informed consent.

## Results

**Figure 1:**
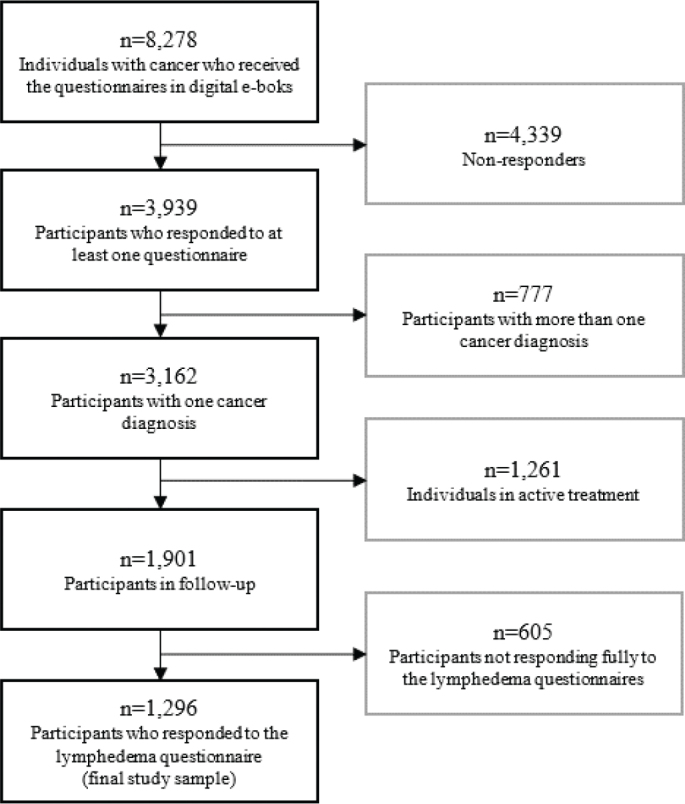
Flowchart for inclusion and exclusion of participants.

At the time of distributing the questionnaire, 8,278 patients were receiving care at our department and were invited to participate, of whom 3,939 completed at least one questionnaire. A total of 1,901 patients were survivors (i.e. in follow-up or hormone treatment) of bladder, colon, breast, gynecological, head/neck, kidney, prostate, or testicular cancer and thus received the lymphedema symptom items. A total of 1,296 completed these items and were included in the current analysis (Figure 1). There were no differences between responders and non-responders of the lymphedema items. In the parent study, there was no difference in sex, but a minimal difference in age [OR 1.00, 95% CI 1.00–1.01] between responders and non-responders, and non-responders were more likely to have a diagnosis of male genital cancer [OR 0.75, 95% CI 0.61–0.93].

### Participants’ characteristics

The participants had a median age of 60 years [IQR 50–70], and 70% were women (*n* = 907). Among the participants, 152 (12%) had comorbidities, 526 (41%) experienced moderate to severe pain interference, and 151 (12%) had depression (either mild, moderate or severe). The median time since diagnosis was 4 years [IQR 2 – 6], and the median BMI was 25 kg/m^2^ [IQR 23 – 28]. Patients with breast cancer represented nearly half of the sample (*n* = 627, 48%), followed by testicular cancer (*n* = 221, 17%), gynecological cancer (*n* = 211, 16%), and head/neck cancer (*n* = 138, 11%). The most commonly reported treatments for cancer were surgery (*n* = 1,128, 87%), chemotherapy (*n* = 888, 69%), and radiation therapy (*n* = 798, 62%), and *n* = 654 (50%) received both surgery and radiation therapy (Table 1).

**Table 1:** Characteristics of participants.

Participants’ characteristics	*N* = 1,296
**Age, years**, median [25% – 75%]	60 [50–70]
**Sex**, *n* (%)	
Female	907 (70%)
Male	389 (30%)
**Body Mass Index, kg/m^2^**, median [25% – 75%]	25 [23–28]
**Charlson Comorbidity Index**, *n* (%)	
No comorbidity	1,144 (88%)
Moderate comorbidity	141 (11%)
High comorbidity	11 (0.8%)
**Depression**, *n* (%)	
No depression	1,142 (88%)
Mild depression	62 (5%)
Moderate Depression	30 (2%)
Severe Depression	59 (5%)
**Pain interference**, *n* (%)	
None/Mild	765 (59%)
Moderate	66 (5%)
Severe	460 (36%)
**Health-related quality of life,^1^** median [25% – 75%]	
Physical functioning	93 [80–100]
Role functioning	100 [67–100]
Emotional functioning	92 [67–100]
Cognitive functioning	83 [67–100]
Social functioning	100 [67–100]
Global Health Status/QoL	89 [78–96]
**Cancer diagnosis**, *n* (%)	
Bladder	26 (2%)
Breast	627 (48%)
Colon	56 (4%)
Gynecological	211 (16%)
Head/Neck	138 (11%)
Kidney	2 (0.2%)
Prostate	15 (1%)
Testicular	221 (17%)
**Time since diagnosis, years**, median [25% – 75%]	4 [2–6]
**Surgery, yes**, *n* (%)	1,128 (87%)
**Chemotherapy;***n* (%)	
Never on treatment	406 (31%)
Previously on treatment	888 (69%)
**Radiation therapy**, *n* (%)	
Never on treatment	498 (38%)
Previously on treatment	798 (62%)
**Hormone therapy**, *n* (%)	
Never on treatment	1,064 (82%)
Previously on treatment	228 (18%)
**Immunotherapy**, *n* (%)	
Never on treatment	1,147 (89%)
Previously on treatment	45 (4%)
Do not know	100 (8%)
**Surgery and radiation therapy, yes**, *n* (%)	654 (50%)

1 Selected sub-scales from The European Organization for Research and Treatment of Cancer Quality of Life Questionnaire (EORTC-QLQ-C30).

### Prevalence and severity of lymphedema symptoms

Every third participant (*n* = 397, 31%) reported lymphedema symptoms. Of these, 62% (*n* = 245) reported mild, and 38% (*n* = 152) reported moderate to severe symptoms. The highest prevalence was observed in gynecological cancer (59%), followed by head/neck (41%), breast (21%), and testicular cancer (19%). Moderate to severe symptoms were reported by 31%, 14%, 6%, and 5% of survivors of gynecological, head/neck, breast, and testicular cancer, respectively. For cancers with fewer than 100 survey responses per cancer type, the prevalence of lymphedema symptoms was 46% in bladder cancer (*n* = 12 of 26), 36% in colon cancer (*n* = 20 of 56), and 67% in prostate cancer (*n* = 10 of 15) (Table 2). Symptoms of lymphedema were most frequently reported by participants who were within 3 years after diagnosis (Supplementary Fig 1).

**Table 2:** Prevalence and severity of lymphedema symptoms across cancer diagnoses.

Cancer diagnosis	Participants *n* = 1,296	Prevalence of symptoms		Severity of lymphedema symptoms	
		No *n* = 899	Yes *n* = 397	Mild *n* = 245	Moderate/severe *n* = 152
**Bladder**	26 (2%)	14 (54%)	12 (46%)	9 (35%)	3 (12%)
**Breast**	627 (48%)	496 (79%)	131 (21%)	92 (15%)	39 (6%)
**Colon**	56 (4%)	36 (64%)	20 (36%)	14 (25%)	6 (11%)
**Gynecological**	211 (16%)	86 (41%)	125 (59%)	59 (28%)	66 (31%)
**Head/neck**	138 (11%)	82 (59%)	56 (41%)	37 (27%)	19 (14%)
**Kidney**	2 (0.2%)	< 5	< 5	< 5	< 5
**Prostate**	15 (1%)	5 (33%)	10 (67%)	2 (13%)	8 (53%)
**Testicular**	221 (17%)	180 (81%)	41 (19%)	30 (14%)	11 (5%)

### Variables associated with lymphedema symptoms

Gynecological, head/neck, breast and testicular cancers each had more than 100 participants and were included in the analysis. Having comorbidities was associated with significantly higher odds of moderate to severe lymphedema symptoms compared to having no comorbidities for participants with breast cancer (OR = 3.09, 95% CI 1.37 to 6.47) and testicular cancer (OR = 5.25, 95% CI 1.06 to 20.6). Patients with breast cancer who were obese (BMI > 30) had significantly higher odds of having moderate to severe lymphedema symptoms (OR = 2.35, 95% CI 1.03 to 5.16) compared to those with normal BMI (BMI ≥ 18.5 to 25). Participants with gynecological cancer who received both surgery and radiation therapy also had significantly higher odds of moderate to severe lymphedema symptoms compared to those who had undergone surgery only (OR = 3.05, 95% CI 1.50 to 6.32). Finally, longer time since diagnosis (years) significantly reduced the odds of moderate to severe lymphedema symptoms for participants with head/neck cancer (OR = 0.62, 95% CI 0.40 to 0.90) (Table 3).

**Table 3:** Sociodemographic and clinical factors and their association with moderate/severe lymphedema symptoms.

Characteristics		Breast					Gynecological					Testicular					Head/Neck			

	*N*	Cases	OR	95% CI	*p*	*N*	Cases	OR	95% CI	*p*	*N*	Cases	OR	95% CI	*p*	*N*	Cases	OR	95% CI	*p*
**Age (per 10 years)**	627		0.89	0.68, 1.17	0.4	211		0.98	0.79, 1.21	0.8	221		1.36	0.85, 2.18	0.2	138		1.21	0.76, 2.00	0.4
**Body Mass Index, kg/m^2^**	623					209					221					138				
Normal 18.5 to 25	315	17	-	-		82	23	-	-		98	3	-	-		75	12	-	-	
Under-weight <18.5	18	0	0.00		-	5	0	0.00			2	0	0.00			5	1	1.31	0.06, 9.89	0.8
Overweight 25 to 30	197	11	1.04	0.46, 2.24	>0.9	67	20	1.09	0.53, 2.22	0.8	86	4	1.54	0.33, 8.03	0.6	43	5	0.69	0.21, 2.02	0.5
Obese > 30	93	11	2.35	1.03, 5.16	0.035	55	21	1.58	0.76, 3.29	0.2	35	4	4.09	0.86, 21.7	0.075	15	1	0.38	0.02, 2.15	0.4
**Charlson Comorbidity Index**	627					211					221					138				
No comorbidity	558	29	-	-		181	57	-	-		204	8	-	-		124	17	-	-	
Comorbidity	69	10	3.09	1.37, 6.47	0.004	30	9	0.93	0.38, 2.11	0.9	17	3	5.25	1.06, 20.6	0.023	14	2	1.05	0.15, 4.31	>0.9
**Time since diagnosis, years**	590		0.96	0.87, 1.04	0.4	197		1.02	0.94, 1.11	0.6	208		1.05	0.92, 1.14	0.4	124		0.62	0.40, 0.90	0.021
**Surgery**	627					211					221					138				
No	4	0	-	-		70	16	-	-		5	1	-	-		66	8	-	-	
Yes	623	39	0.00		-	141	50	1.85	0.98, 3.65	0.065	216	10	0.19	0.03, 3.98	0.2	72	11	1.31	0.49, 3.60	0.6
**Chemotherapy**	626					210					221					138				
Never on treatment	207	15	-	-		16	7	-	-		127	4	-	-		40	6	-	-	
Previously on treatment	419	24	0.78	0.40, 1.55	0.5	194	59	0.56	0.20, 1.64	0.3	94	7	2.47	0.72, 9.69	0.2	98	13	0.87	0.31, 2.64	0.8
**Radiation therapy**	627					211					221					138				
Never on treatment	143	5	-	-		86	22	-	-		199	10	-	-		7	1	-	-	
Previously on treatment	484	34	2.09	0.87, 6.17	0.13	125	44	1.58	0.87, 2.94	0.14	22	1	0.90	0.05, 5.06	>0.9	131	18	0.96	0.15, 18.6	>0.9
**Hormone therapy**	625					209					221					138				
Never on treatment	406	26	-	-		208	65	-	-		220	11	-	-		137	19	-	-	
Previously on treatment	219	12	0.85	0.40, 1.68	0.6	1	1	0.00		-	1	0	0.00		-	1	0	0.00		-
**Immunotherapy**	625					209					221					138				
Never on treatment	534	36	-	-		192	62	-	-		214	10	-	-		130	19	-	-	
Previously on treatment	26	0	0.00		-	1	0	0.00	-	-	1	0	0.00		-	4	0	0.00		-
Do not know	65	2	0.44	0.07, 1.49	0.3	16	4	0.70	0.19, 2.10	0.5	6	1	4.08	0.20, 28.7	0.2	4	0	0.00		-
**Combined treatment**	623					141					216					72				
Surgery alone	135	5	-	-		61	20	-	-		186	9	-	-		5	1	-	-	
Surgery and radiation therapy	449	34	2.04	0.86, 6.05	0.14	30	30	3.05	1.50, 6.32	0.002	20	1	1.03	0.05, 5.93	>0.9	56	10	0.89	0.13, 18.1	>0.9

OR: odds ratio; CI: confidence interval; Age (per 10 years): Coefficients reflect the change in the dependent variable for every 10-year increase in age.

No significant associations were found between age, surgery alone, chemotherapy, radiation therapy, and lymphedema symptoms across the included cancer diagnoses. Odds ratios for immunotherapy could not be calculated due to insufficient cases who reported to have received immunotherapy (Table 3).

### The association of depression, pain interference, and HRQoL, with lymphedema symptoms

For participants with moderate to severe lymphedema symptoms, there was a statistically significant association with higher depression and pain interference scores compared to those with no or mild lymphedema symptoms. Moderate to severe lymphedema symptoms had a significant negative association with lower HRQoL across multiple scales, including physical, role, cognitive, and emotional functioning, as well as global health status/QoL, compared to those with no or mild symptoms (Table 4). These associations remained significant after adjustment for sociodemographic and clinical variables (e.g. depression: MD = 4.5, 95% CI 3.0 to 6.0; pain interference: MD = 1.9, 95% CI 1.2 to 2.6; physical: MD = −12.7, 95% CI −22.0 to −3.3; role: MD = −27.0, 95% CI −35.1 to −18.8; cognitive: MD = −15.6, 95% CI −25.6 to −6.1; emotional: MD = −11.9, 95% CI −15.9 to −7.9; global health status/QoL: MD = −10.5, 95% CI −13.0 to −8.0).

**Table 4:** Association of depression, pain interference and health-related quality of life with moderate to severe symptoms of lymphedema.

Outcome variables	Difference of means (Welch T-test)			Adjusted Model I			Adjusted Model II		
	Difference in means	95% CI^1^	*p*	Adjusted difference in means	95% CI^1^	*p*	Adjusted difference in means	95% CI^1^	*p*
EORTC-QLQ-C30									
Physical functioning	−17.9	−21.9, −13.9	< 0.001	−16.4	−19.4, −13.4	< 0.001	−12.7	−22.0, −3.3	0.008
Role functioning	−20.6	−26.0, −15.2	< 0.001	−16.7	−25.8, −7.6	< 0.001	−27.0	−35.1, −18.8	< 0.001
Social functioning	−12.6	−17.1, −8.1	< 0.001	−9.4	−15.2, −3.5	0.002	−6.7	−16.5, 3.3	0.188
Cognitive functioning	−10.0	−14.6, −5.6	< 0.001	−9.7	−13.6, −5.8	< 0.001	−15.6	−25.0, −6.1	< 0.001
Emotional functioning	−9.5	−13.6, −5.3	< 0.001	−12.4	−16.2, −8.5	< 0.001	−11.9	−15.9, −7.9	< 0.001
Global Health Status/QoL	−11.8	−14.6, −9.0	< 0.001	−10.7	−13.1, −8.3	< 0.001	−10.5	−13.0, −8.0	< 0.001
Depression	4.9	3.3, 6.5	< 0.001	4.6	3.1, 6.0	< 0.001	4.5	3.0, 6.0	< 0.001
Pain Interference	1.5	1.1, 2.6	< 0.001	2.0	1.3, 2.7	< 0.001	1.9	1.2, 2.6	< 0.001

*Linear regression was conducted for the seven dependent variables and the independent variable presence/absence of moderate/severe lymphedema.

EORTC-QLQ-C30: European Organization for Research and Treatments of Cancer Quality of Life Questionnaire. Depression: Major Depression Inventory. Pain Interference: Brief Pain Inventory.

CI: confidence interval.

Adjusted Model I = Linear regression adjusted for sex, age, BMI, Charlson Comorbidity Index.

Adjusted Model II = Linear regression adjusted for Adjusted model I + time since treatment, treatment (surgery, chemotherapy, radiation therapy, hormone therapy, immunotherapy).

For social functioning, the association did not remain statistically significant in the adjusted model II (MD = −6.7, 95% CI −16.5 to 0.33), suggesting that clinical variables may influence this relationship (Table 4).

### The risk of exceeding HRQoL thresholds of clinical importance

**Figure 2:**
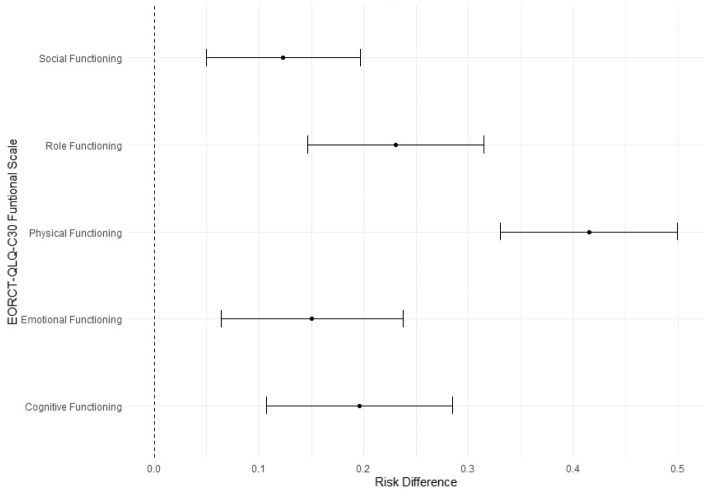
Risk difference of exceeding the threshold for clinical importance of health-related quality of life functioning scales between the presence and absence of moderate/severe symptoms of lymphedema.

Across the five HRQoL functioning scales (physical, role, social, emotional, and cognitive functioning), participants with moderate to severe symptoms of lymphedema had a significantly higher risk of exceeding the threshold for clinical importance compared to those with no or mild symptoms, indicating clinically significant problems (Figure 2). The risk difference ranged from 12% to 41%. The largest difference was observed in the physical functioning scale, where participants with moderate to severe symptoms of lymphedema had a 41% higher risk of exceeding the clinical threshold compared to those with no or mild symptoms.

## Discussion

In this study, we addressed the lack of comparable prevalence estimates for lymphedema symptoms across cancer diagnoses in a diverse sample of cancer survivors in follow-up care, demonstrating a high prevalence of lymphedema symptoms across diagnoses. The highest rates were reported by participants with gynecological cancer (59%), followed by those with head/neck cancer (41%), breast cancer (21%), and, notably, testicular cancer (19%). Overall, 38% of participants with lymphedema symptoms reported moderate to severe symptoms, which were associated with higher scores for depression and pain interference and lower HRQoL scores, compared to participants with no or mild symptoms.

Among women with breast cancer, 21% experienced lymphedema symptoms, aligning with previous estimates of around one in five [21]. Conversely, 59% of women with gynecological cancer experienced symptoms, which is notably higher than the 38% prevalence reported 24 months post-surgery in a prospective study by Hayes et al. with 390 Australian patients [22]. The discrepancy may be due to differences in the PRO measure, as our study defined lymphedema symptoms as swelling, heaviness, or tightness in the legs, groin, and genital region, whereas Hayes et al. focused solely on leg swelling. Additionally, differences in gynecological cancer type, treatment combinations, and cancer stage between the two studies may contribute to the differences observed [22]. In our study, 41% of participants with head/neck cancer reported lymphedema symptoms, compared to 82% in the longitudinal American study (*n* = 83) by Ridner et al. [23]. The higher prevalence in Ridner’s study may be explained by the fact that 70% of their participants had stage IV cancer, as well as using a more comprehensive assessment method, including clinical assessment for lymphedema. Finally, selection bias from convenience sampling may have skewed their results toward patients with more severe symptoms.

A recent systematic review [24] reported lymphedema incidences ranging from 2–74% in breast cancer, 8–45% in gynecological cancers, and 71–90% in head/neck cancers. This variability reflects the challenges in comparing prevalence across studies due to methodological differences such as study design, patient populations, and measurement methods. Many studies rely on selective sampling, for example, including high-risk patients only. Factors like cancer stage, age, and the definition of lymphedema further contribute to variability. In contrast, we included all cancer survivors in follow-up care at our department and thus offer an estimate of the prevalence and severity of lymphedema symptoms across multiple cancer diagnoses, allowing for better comparability and understanding of its extent.

To the best of our knowledge, we are the first to report a 19% prevalence of lymphedema symptoms in men with testicular cancer. Given that testicular cancer predominantly affects younger men, further research is needed to determine whether these symptoms are indicative of clinical lymphedema and their impact on daily life. A qualitative study involving men treated for cancer-related lymphedema highlighted body image concerns, along with physical symptoms such as swollen genitals and urinary difficulties, which severely affected functioning and psychological well-being [6]. Additionally, issues related to sexuality and possible differences in the impact of lymphedema between men and women should be considered [25]. The combination of men’s health-seeking behavior, lack of risk-awareness and inconsistent surveillance could present barriers to early detection and timely management of lymphedema [26–28].

With our findings, we join the chorus of researchers calling for a broader approach to lymphedema care, suggesting that surveillance and awareness should be expanded to include both male and female survivors across various diagnoses to ensure timely care for all at-risk patients. Further longitudinal studies are warranted to confirm and expand upon these findings.

When suggesting surveillance for lymphedema, it is important to consider how to identify the high-risk patients. We identified three factors associated with moderate to severe lymphedema symptoms, but none were present across all cancer diagnoses. Obesity, a recognized risk factor, was associated with lymphedema symptoms in breast cancer, consistent with previous studies [2, 21]. The combination of surgery and radiation therapy was linked to moderate to severe symptoms of lymphedema in participants with gynecological cancers, yet no single treatment modality (surgery, radiation, or chemotherapy) was independently associated with moderate to severe lymphedema symptoms, contrasting existing literature [3, 21, 29], which is likely explained by our small sample size for each cancer diagnosis. Comorbidities were associated with increased odds of reporting lymphedema symptoms in breast and testicular cancers, consistent with previous findings for breast cancer [22, 30], while no previous studies have explored prevalence nor risk factors for lymphedema among survivors of testicular cancer.

Participants with moderate to severe symptoms were significantly more likely to report higher scores for depression and pain interference and lower HRQoL scores. As pain interference is based on a generic measure, the source of pain is unknown, leaving it unclear if it relates to lymphedema symptoms. The association between lymphedema symptoms and depression remains underexplored. A matched case-control study from Portugal linked cancer-related and primary lymphedema to increased depression, suggesting a broader psychological impact of this condition [31]. Our study supports this link across cancer diagnoses, providing new insights into the relationship between lymphedema symptoms and depression.

We found a negative relationship between moderate to severe lymphedema symptoms and HRQoL, consistent with findings from Carter et al.’s prospective study of 768 women with gynecologic cancers, which demonstrated that lymphedema symptoms were significantly associated with lower HRQoL [8]. Similar patterns were reported in a large cross-sectional study of 1,127 women with endometrial cancer [32], as well as a systematic review on lower limb lymphedema among cancer survivors [7]. Furthermore, participants with moderate to severe lymphedema symptoms were at a greater risk of exceeding the clinical thresholds across all HRQoL functioning scales [19]. Physical functioning (i.e. ability to walk, need for rest, and assistance with daily activities) was most affected, with a 41% higher risk of exceeding the threshold, underscoring the impact of lymphedema symptoms on physical capabilities and the need for clinical intervention.

There are some limitations to our study that should be considered. First, the study relied on self-reported lymphedema symptoms without objective validation (e.g. bioimpedance or clinical assessment). However, by focusing on core symptoms – swelling, heaviness, and tightness – the risk of misclassification was reduced. The self-reported symptoms provided valuable insight into how survivors experience lymphedema symptoms in their daily lives. Second, the cross-sectional nature of our study limits our ability to establish causality and temporality. We cannot determine whether lymphedema symptoms were present before the cancer diagnosis or if they developed because of cancer treatment or comorbid conditions. Likewise, we cannot infer causality between lymphedema and associated factors such as depression, pain interference, and HRQoL. However, the strength of this study is its inclusive sampling approach, which minimized selection bias by inviting all patients in follow-up. This inclusive approach provides a more representative estimate of lymphedema symptoms across a diverse population of cancer survivors, enhancing the generalizability of our findings.

## Conclusion

Across cancer survivors in follow-up at our Department of Oncology, symptoms of lymphedema are highly prevalent, and moderate to severe symptoms are associated with higher scores for depression and pain interference and lower HRQoL. These findings highlight the need for increased awareness of lymphedema among both male and female cancer survivors and the importance of directing future research toward better understanding and managing lymphedema as well as implementing procedures for screening, early detection, and treatment among survivors at high risk.

## Author contributions

Conceptualization: GSL, AvH, BSR

Methodology: GSL, AvH, BSR

Analysis: GSL

Accessed and verified the underlying data: GSL, AvH, BSR

Writing (original draft preparation): GSL

Writing (review and editing): GSL, AvH, BSR, CJ

Funding acquisition: Not applicable

All authors had full access to all the data and have final responsibility for the decision to submit for publication.

## Supplementary Material

Prevalence of lymphedema symptoms across cancer diagnoses and association with depression, pain interference and health-related quality of life

## Data Availability

Data is available upon request to the corresponding author.
