# Supplementary material for: Prevalence of lymphedema symptoms across cancer diagnoses and association with depression, pain interference and health-related quality of life
Source: Acta Oncol. 2025 Jan 21;64:42203. doi: 10.2340/1651-226X.2025.42203 (PMC11776255; doi:10.2340/1651-226X.2025.42203)

Supplementary material has been published as submitted. It has not been copyedited, or typeset by Acta Oncologica

Supplementary file 1: Distribution of lymphedema symptoms by years since diagnosis

A) Distribution of the presence of any lymphedema symptoms by years since diagnosis

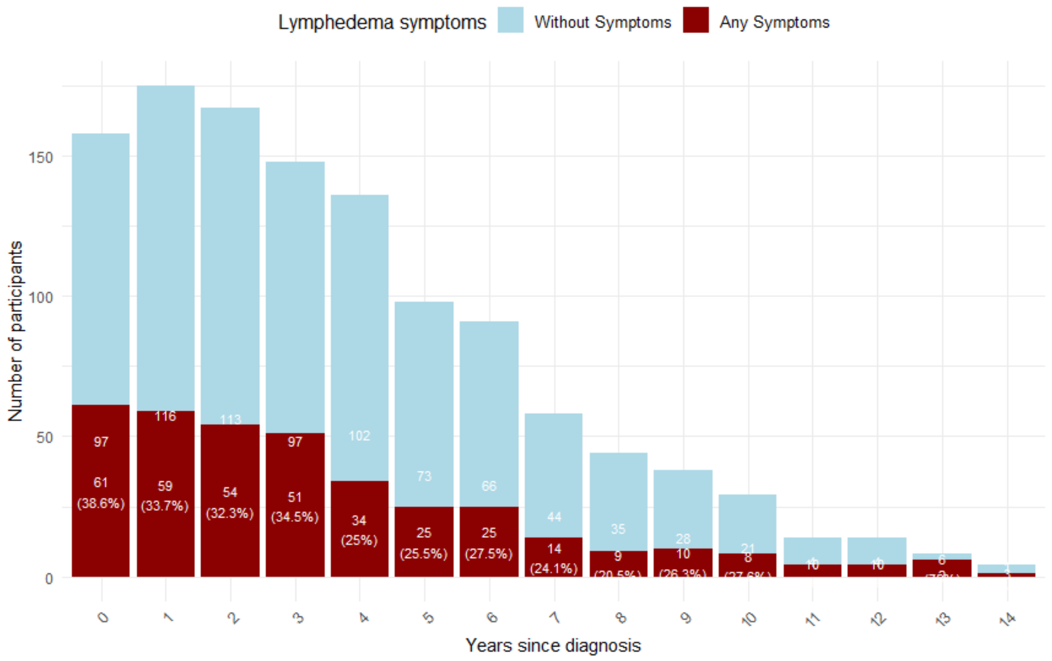

B) Distribution of moderate to severe lymphedema symptoms by years since diagnosis

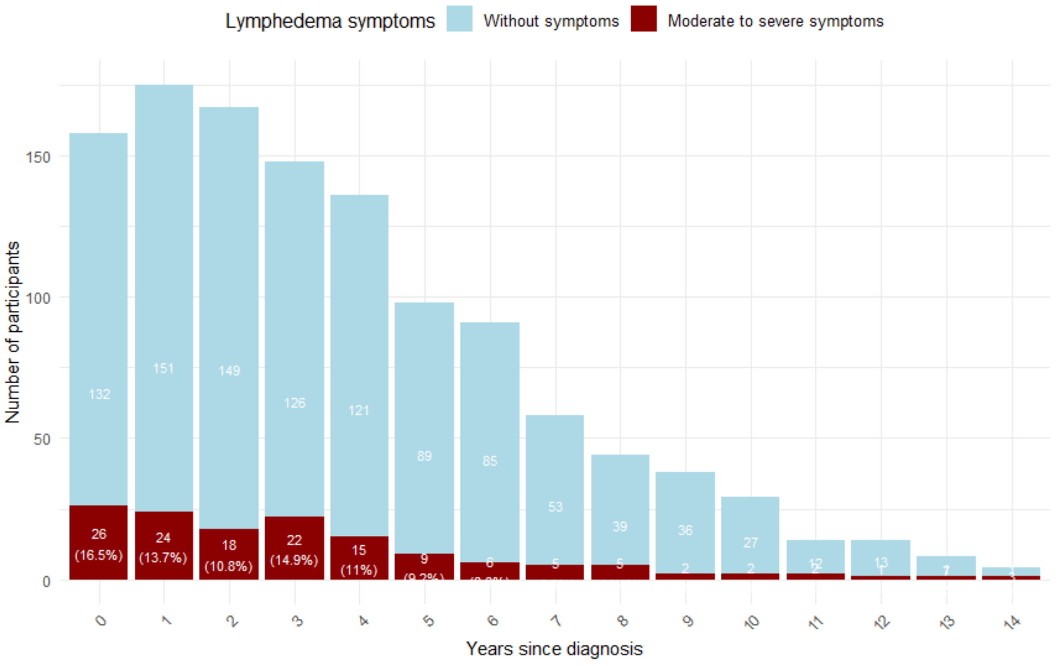

Supplement: Prevalence of lymphedema symptoms across cancer diagnoses and association with depression, pain interference and health-related quality of life [file AO-64-42203-s1.pdf]
